# The m^6^A Reader YTHDF1 Promotes Lung Carcinoma Progression via Regulating Ferritin Mediate Ferroptosis in an m^6^A-Dependent Manner

**DOI:** 10.3390/ph16020185

**Published:** 2023-01-25

**Authors:** Hongtao Diao, Huiling Tan, Yaju Hu, Ruonan Wang, Pingdong Cai, Bingying Huang, Xiaoqi Shao, Meiling Yan, Chuntong Yin, Yue Zhang

**Affiliations:** 1 Guangdong Metabolic Diseases Research Center of Integrated Chinese and Western Medicine, Guangdong TCM Key Laboratory for Metabolic Diseases, Guangdong Pharmaceutical University, Guangzhou 510006, China; 2 Key Laboratory of Glucolipid Metabolic Disorder, Ministry of Education of China, Guangzhou 510006, China; 3 Department of Thoracic Surgery, The First Affiliated Hospital of Anhui Medical University, Hefei 230022, China

**Keywords:** lung cancer, YTHDF1, N6-methyladenosine (m^6^A) modification, ferroptosis, ferritin

## Abstract

N6-methyladenosine (m^6^A) plays a significant role as an epigenetic mechanism, which is involved in various cancers’ progress via regulating mRNA modification. As a crucial m^6^A “reader”, YTHDF1 is able to alter m^6^A-modified mRNA and promote the protein translation process in multiple cancers. However, the role of YTHDF1 in lung cancer has not been fully investigated. This study focuses on elucidating the function of YTHDF1 in the development of lung cancer and its underlying mechanism. We demonstrated that YTHDF1 was highly expressed in lung carcinoma progression; then, the loss of function experiments in lung cell lines confirmed that knockdown of YTHDF1 suppressed cell proliferation, migration and invasion and induced ferroptosis of lung cancer cells. Further functional assays showed that ferritin (FTH) was identified as the key target of YTHDF1 in lung cancer cells. Furthermore, the overexpression of ferritin in YTHDF1-depleted cells partially restored lung cancer cell suppression. Collectively, our data suggested that the upregulation of YTHDF1 promotes lung cancer carcinogenesis by accelerating ferritin translation in an m^6^A-dependent manner. We hope that our findings may provide a new target for lung cancer diagnosis and treatment.

## 1. Introduction

Lung cancer continues to be the leading cause of cancer death around the world in recent years [1]. Although, treatment for lung carcinoma is mainly done through surgical intervention, chemotherapy or immunotherapy [2,3]. However, due to malignancy recurrence and drug treatment failure, the overall survival rates of lung carcinoma patients to chemotherapy remains poor [4]. Therefore, it is urgent for us to understand the pathogenesis of lung cancer and discover a novel and effective therapeutic target.

N6-methyladenosine (m^6^A) is the most common post-transcriptional modification of mRNA in eukaryotes [5]. It can participate in stem cell reprogramming, cell proliferation, and the occurrence and development of various diseases by dynamically regulating the alternative splicing, stability and translation efficiency [6,7]. The m^6^A regulators include m^6^A “writers”, “erasers” and “readers”, and can dynamically recognize and regulate related proteins [8]. Cumulative studies have proved that m^6^A modification affects diverse malignant cancer initiation and progression through multiple mechanisms, such as hepatocellular cancer [9,10], colorectal cancer [11], bladder cancer [12] and breast cancer [13]. Notably, YTHDF1 is a major m^6^A reader, which targets mRNAs translation machinery. It affects protein translation and functions as a crucial initiating factor in multiple pathogeneses [14]. A previous study indicated that YTHDF1 upregulated autophagy-related genes to promote hepatocellular carcinoma (HCC) progression [15]. However, due to the complexity of lung tumorigenesis and progression, the molecular mechanisms for how the m^6^A reader YTHDF1 could regulate gene translation and expression have not yet been described.

Ferroptosis is a brand-new form of cell death, which is characterized by iron-dependent lipid peroxidation production [16]. The excessive accumulation of lipids and reactive oxygen species (ROS) and the missing activity of Glutathione peroxidase 4 (GPX4) have been shown to induce ferroptosis [17]. It is well accepted that ferroptosis and iron-metabolism are essential to regulate hepatocellular tumorigenesis and predict clinical diagnosis, prognosis and immune microenvironment [18]. Of note, ferritin is composed of a light subunit (FTL) and a heavy subunit (FTH), and the latter is a well-known regulator of cellular iron uptake and storage [19]. The degradation of ferritin would cause the transport interruption of normal free iron level and may cause excessive accumulation of iron [20]. High levels of free iron within the cells are extremely harmful, and it can cause severe oxidative damage via the Fenton reaction, leading to reactive oxygen species (ROS) generation [21], eventually leading to cell death.

Despite some previous studies also confirming the connection between m^6^A modification and ferroptosis in cancer development, it is previously reported that YTHDF1 is bound with iron metabolism and highly expressed in hypopharyngeal squamous cells (HPSCCs) by regulating TFRC expression in an m^6^A-dependent mechanism [22]. However, the detailed regulatory pathways and crucial regulators of ferroptosis during lung adenocarcinoma progression remain to be explored. Thus, we aim to address the consequences of the m^6^A modification reader YTHDF1 in lung cancer and speculate on the related mechanism.

In the present study, we demonstrated that YTHDF1 is upregulated in lung carcinoma patients. Further molecular mechanism investigation indicated that interfering with YTHDF1 expression restrains cell proliferation, migration and invasion, ultimately promoting ferroptosis. Moreover, we also found that YTHDF1-mediated m^6^A modification promotes lung tumorigenesis by regulating FTH-mediated ferroptosis. Furthermore, FTH overexpression could significantly rescue the tumorigenic effect of YTHDF1 induction. Overall, our study revealed that the YTHDF1–m^6^A–FTH axis participates in the progression of lung adenocarcinoma, suggesting the pathway axis might be a novel therapeutic target for lung adenocarcinoma treatment.

## 2. Results

### 2.1. YTHDF1 Is Frequently Amplified in Lung Carcinoma

**Figure 1:**
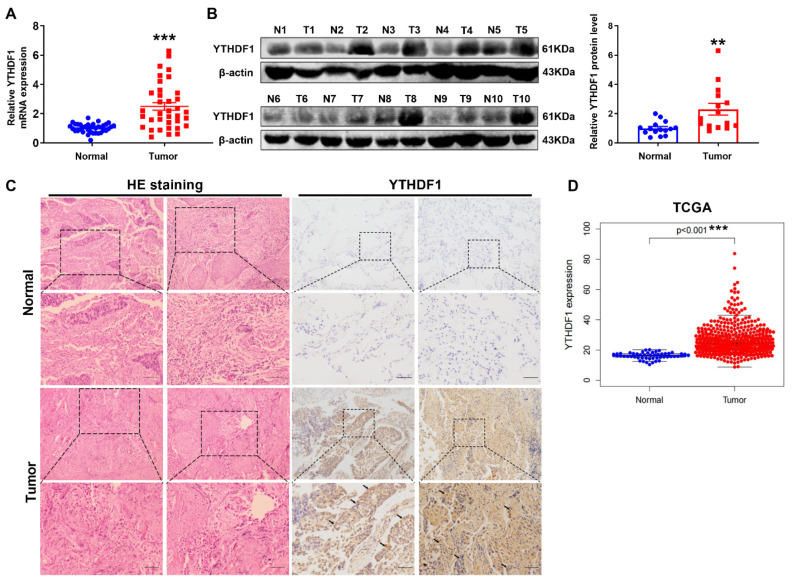
YTHDF1 is significantly expressed in lung cancer. (**A**) The mRNA level of YTHDF1 was analyzed by qRT-PCR in lung cancer tissues and adjacent normal tissues. (**B**) The protein level of YTHDF1 was detected in clinical lung cancer tissues (T) compared with corresponding adjacent normal lung tissues (N). (**C**) Pathological changes of tumor were observed in HE staining. IHC staining of YTHDF1 in lung cancer tissues at 10 × magnification. Scale bar, 100 μm. (**D**) YTHDF1 expression in lung cancer according to TCGA dataset. ^**^ *p* < 0.01 and ^***^ *p* < 0.001.

To investigate the expression and clinical correlation of the m^6^A reader YTHDF1 in lung cancer, mRNA expression of YTHDF1 was analyzed between lung cancer tissues and adjacent normal lung tissues (negative control). We found that YTHDF1 was significantly highly expressed in lung tissue samples compared with normal lung tissues (Figure 1A). Meanwhile, the expression of YTHDF1 protein was relatively high in lung tissues (Figure 1B). Pathological changes of tumor were observed in HE staining, which showed the classic pathological features of adjacent normal tissues and tumor tissues. Further results of immunohistochemical analysis revealed that YTHDF1 level was remarkably upregulated in lung tissues (Figure 1C). In addition, the expression of YTHDF1 was analyzed by using the TCGA dataset, which indicated that YTHDF1 was frequently upregulated in lung cancer (Figure 1D). These data demonstrated that the m^6^A reader YTHDF1 is upregulated in lung cancer and may be connected with lung cancer tumorigenesis and progression.

### 2.2. YTHDF1 Promotes Lung Cancer Cells Proliferation, Migration and Invasion

**Figure 2:**
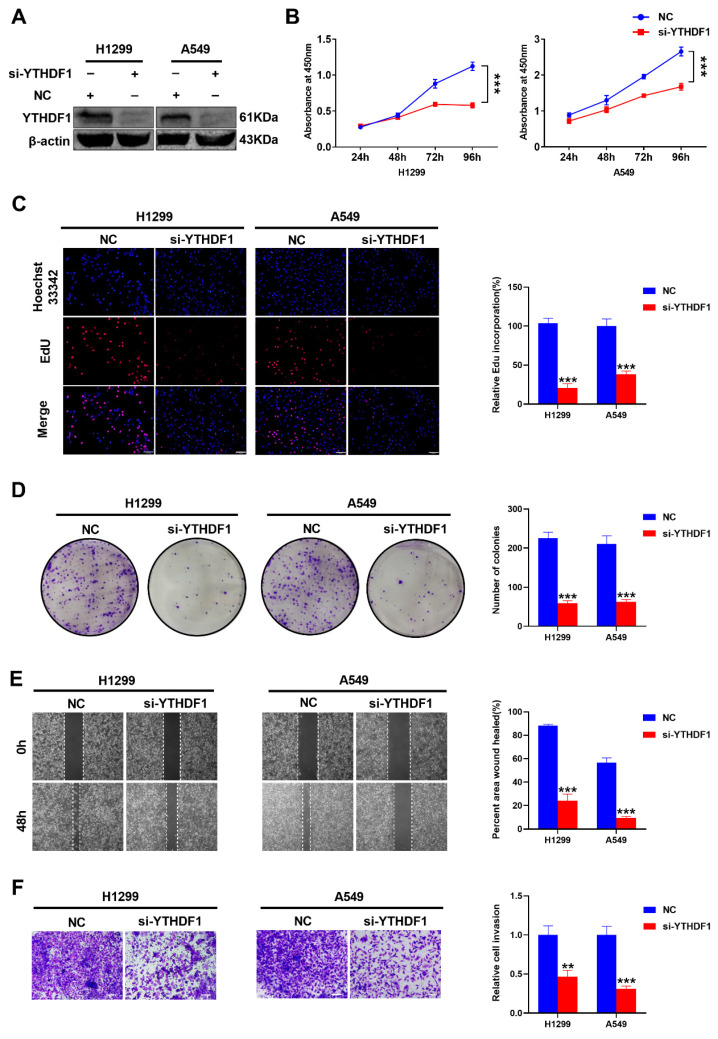
YTHDF1 promotes lung cancer cell proliferation, metastasis and invasion. (**A**) Western blot analysis of transfection with si-YTHDF1 in H1299 and A549 cells. (**B**–**D**) CCK-8 assays, Edu assays and colony formation assays for H1299 and A549 cells with YTHDF1 knockdown. Scale bar, 100 μm. (**E**,**F**) Wound-healing assays and Transwell Matrigel invasion assays for H1299 and A549 cells with YTHDF1 downregulation. Scale bar, 100 μm. ^**^ *p* < 0.01 and ^***^ *p* < 0.001.

In order to explore the biological role of YTHDF1 in lung cancer, YTHDF1 was knocked down in human lung adenocarcinoma cell lines. First, siRNAs were used to silence YTHDF1 in H1299 and A549 cells (Fuheng Biotechnology Co., Ltd., Shanghai, China). Western blot analysis showed that YTHDF1 was knocked down (Figure 2A). Then, the effect of YTHDF1 on cell proliferation, migration and invasion was examined. The results of the CCK-8 assay and colony formation assay revealed that, compared with the negative control (NC), YTHDF1 depletion suppressed the growth ability of lung cancer cells (Figure 2B,D). Similarly, the Edu assay also demonstrated that the knockdown of YTHDF1 could inhibit cell growth (Figure 2C). Next, the wound-healing and Matrigel-coated Transwell assays were used to assess whether YTHDF1 could affect the migration and invasion of lung cancer cells, indicating that YTHDF1 knockdown markedly reduced the migration and invasion of H1299 and A549 cells in vitro (Figure 2E,F). All in all, these data suggested that YTHDF1 promotes lung cancer cell proliferation, migration and invasion in vitro and plays an oncogenic role in lung carcinoma.

### 2.3. Knockdown of YTHDF1 Increased Intracellular Iron Levels and Promoted Lung Cancer Cell Ferroptosis

**Figure 3:**
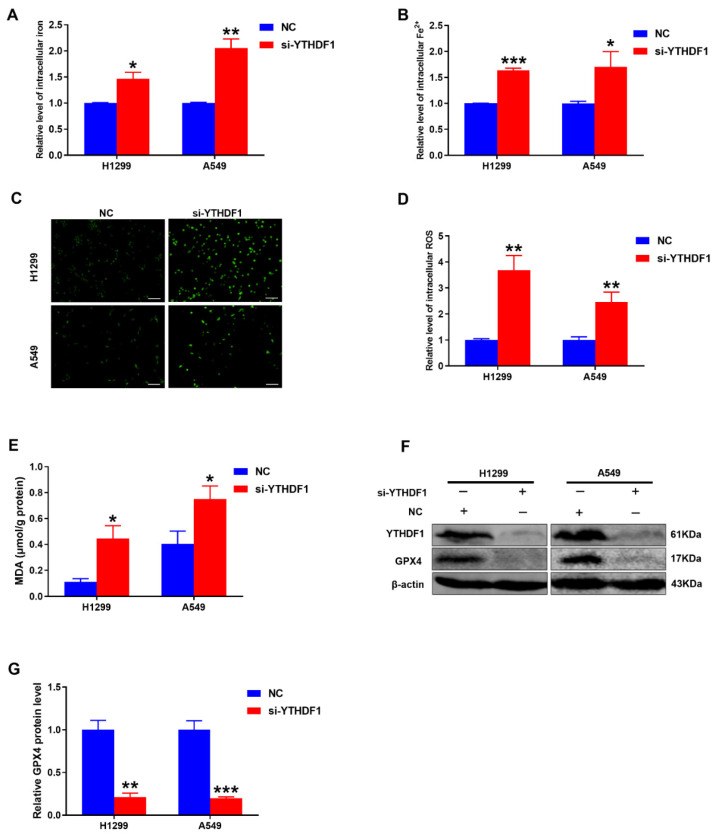
YTHDF1 knockdown induced dysregulated iron metabolism and promoted ferroptosis of lung cancer cells. (**A**,**B**) Intracellular iron level and Fe^2+^ level in H1299 and A549 cells after transfection with si-YTHDF1 at 24 h. (**C**) Representative fluorescence images of H1299 and A549 cells stained with DCFH-DA to measure ROS production. Scale bar, 100 μm. (**D**) Statistical analysis of the green fluorescence about ROS. (**E**) MDA level was detected in YTHDF1 depletion lung cancer cell lines. (**F**) Western blot analysis showed the effect of YTHDF1 knockdown on GPX4 in lung cancer cell lines. (**G**) Statistical analysis for the result of GPX4 protein assays in (**E**). ^*^ *p* < 0.05; ^**^ *p* < 0.01 and ^***^ *p* < 0.001.

Accumulated studies have confirmed that RNA m^6^A modification is involved in the regulation of various cancer ferroptosis [23,24]. We next explored whether YTHDF1 depletion would induce ferroptosis in lung cancer cells. The results showed that intracellular total iron and Fe^2+^ levels were significantly increased in si-YTHDF1-infected cells compared to the controls (Figure 3A,B). What is more, the productions of ROS and malondialdehyde (MDA) were well-established indicators in ferroptosis [25]. As expected, the MDA level was greatly increased after YTHDF1 silencing (Figure 3E) compared with the control group (Figure 3C). ROS production was increased in the YTHDF1 knockdown group (Figure 3D). More importantly, the crucial ferroptosis-related protein GPX4 level was decreased in the YTHDF1 silencing group compared to the NC group (Figure 3F,G). These results strongly suggested that YTHDF1 depletion increased intracellular iron levels and promoted lung cancer cell ferroptosis.

### 2.4. YTHDF1 Regulated FTH Expression by Enhancing Its Stability through an m^6^A-Dependent Manner in Lung Cancer

**Figure 4:**
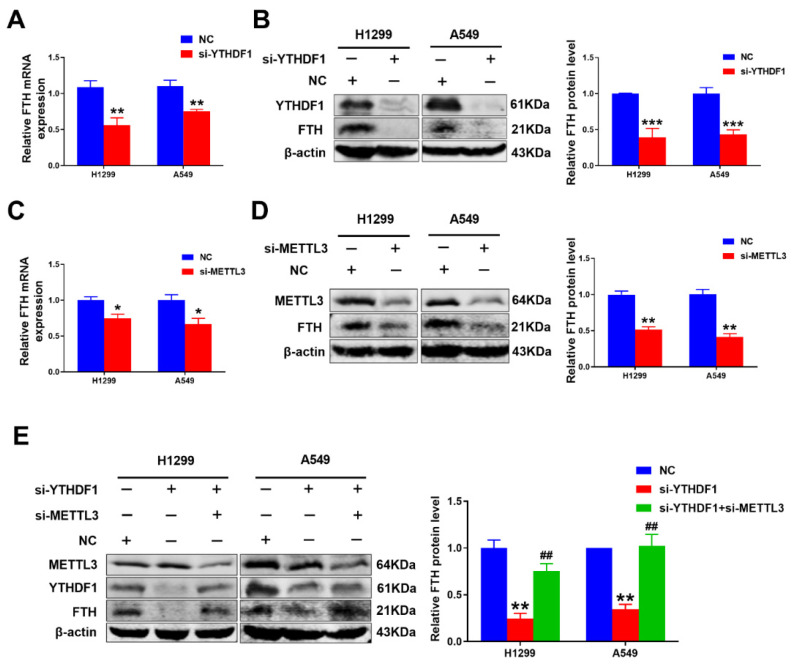
YTHDF1 can positively regulate FTH expression in an m^6^A-dependent manner. (**A**,**B**) Relative mRNA level and protein level were measured by qRT-PCR and Western blot of FTH in YTHDF1 knockdown H1299 and A549 cells. (**C**,**D**) The relative mRNA expression and protein level were determined by qRT-PCR and Western blot of FTH after METTL3 knockdown in H1299 and A549 cells. (**E**) The protein level of FTH revealed by Western blot in YTHDF1 knockdown lung cancer cells with METTL3 depression, compared with negative control lung cancer cells. ^*^ *p* < 0.05; ^**^ *p* < 0.01; ^***^ *p* < 0.001; and ^##^ *p* < 0.01.

It is generally known that m^6^A modification would recognize that selective binding proteins affect the translation and degradation of RNA [26], but the interaction between m^6^A modification and ferroptosis in lung cancer and the specific mechanism remains unknown. Recent research demonstrated that ferritin (FTH) was a key ferroptosis regulator and its level affected the susceptibility to ferroptosis [27,28]. Thus, we investigated whether the loss of YTHDF1 could affect the expression of FTH. With qRT-PCR detection, we found that si-YTHDF1 decreased the mRNA expression of FTH; in a Western blot, the FTH protein level was notably downregulated after YTHDF1 silencing compared to controls (Figure 4A,B). As a previous study reported, METTL3 could cooperate with the m^6^A reader YTHDF1 to regulate related-target transcript stability [29,30,31]. Consequently, we knocked down METTL3 by siRNA transfection in lung cancer cells and then analyzed the expression levels of FTH in H1299 and A549 cell lines. The data showed that FTH expression was obviously reduced in METTL3 knockdown cells (Figure 4C,D). Furthermore, the downregulation of METTL3 could markedly counteract the effects of YTHDF1 depletion on the expression of FTH expression at protein levels (Figure 4E,F), which indicated that the potential role of YTHDF1 in FTH modulation depended on METTL3-medicated m^6^A modification. Taken together, our results demonstrated that YTHDF1 depletion decreased the expression of FTH in an m^6^A-dependent manner.

### 2.5. FTH Overexpression Reversed the Tumor Proliferation, Migration and Invasion in YTHDF1 Depletion Lung Cancer Cells

**Figure 5:**
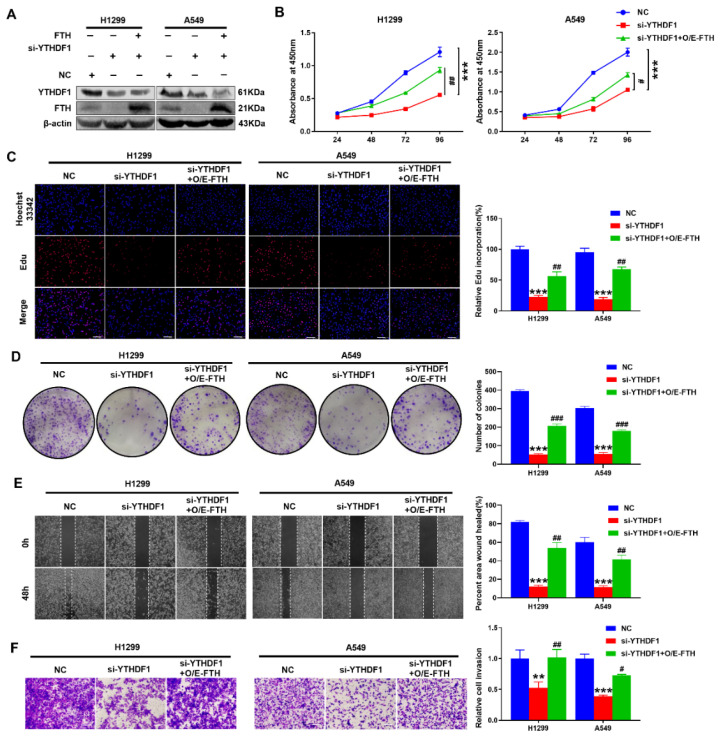
FTH overexpression effectively altered YTHDF1 knockdown-induced inhibition of lung cancer cell progression. (**A**) Western blot analysis of the transfection efficiency of FTH in lung cancer cells. (**B**–**D**) CCK-8 assay, Edu incorporation assay and colony formation assay of lung cancer cells transfected with FTH and si-YTHDF1, respectively or corporately. (**E**,**F**) Wound-healing assay and Transwell Matrigel invasion assay of lung cancer cells transfected with FTH and si-YTHDF1, respectively or corporately. Scale bar, 100 μm. ns, not significant; ^**^ *p* < 0.01; ^***^ *p* < 0.001; ^#^ *p* < 0.05; ^##^ *p* < 0.01; and ^###^ *p* < 0.001.

**Figure 6:**
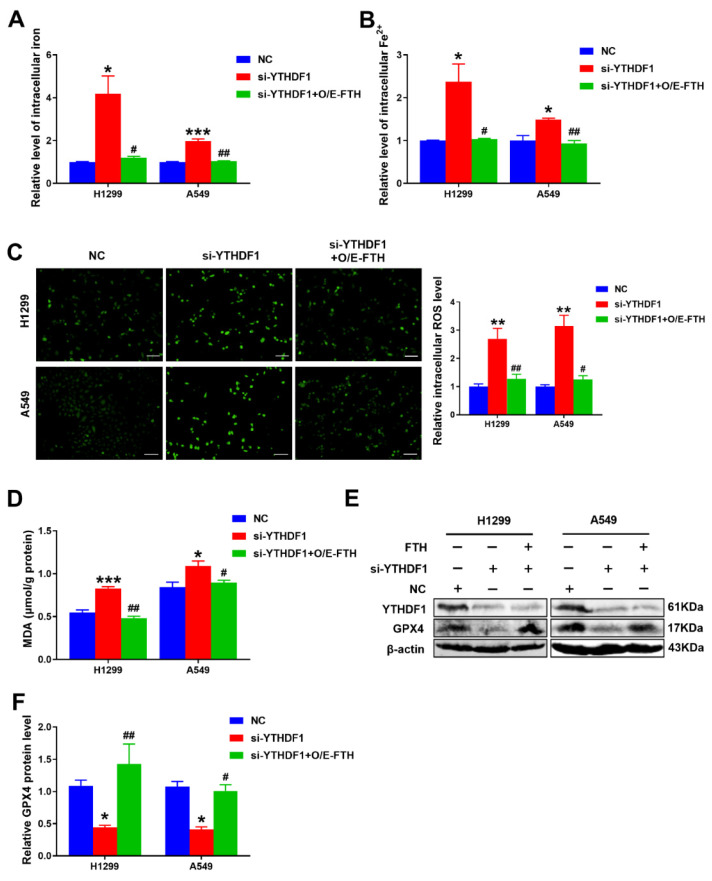
YTHDF1 knockdown induced FTH-mediated ferroptosis. (**A**,**B**) An intracellular iron assay showed the effect of FTH overexpression on intracellular total iron and Fe2+ levels. (**C**) Intracellular ROS level was measured by DFCH-DA fluorescence with transfection of NC siRNA, YTHDF1 siRNA or overexpressed FTH in H1299 and A549 cells. Scale bar, 100 μm. (**D**) An MDA assay showed the impact of FTH overexpressed in YTHDF1 knockdown cells. (**E**) The protein level showed by a Western blot of GPX4 upon the YTHDF1 depletion or the FTH overexpression in lung cancer cell lines. (**F**) Statistical analysis for the result of GPX4 protein assays in (**E**). ^*^ *p* < 0.05; ^**^ *p* < 0.01; ^***^ *p* < 0.001; ^#^ *p* < 0.05; and ^##^ *p* < 0.01.

The major mechanism through which m^6^A exerts its role is mainly dependent on m^6^A-binding proteins [32]. Therefore, we evaluated whether FTH participates with YTHDF1 in promoting lung carcinoma cell proliferation, migration and invasion in our next study. To further examine the role of FTH in lung cancer cells, a Western blot showed that FTH was overexpressed in both H1299 and A549 cells (Figure 5A). CCK-8 and Edu assays found that the proliferation of H1299 and A549 cells were boosted after FTH overexpression (Figure 5B,C). A colony formation assay also revealed that the overexpression of FTH markedly promoted the colony formation ability of H1299 and A549 cells (Figure 5D). In addition, the results of wound-healing and Matrigel-coated Transwell assays were vastly increased in the overexpression of FTH cells compared with YTHDF1-silencing cells (Figure 6E,F). All of these results verified that YTHDF1 promotes lung cancer cell proliferation, migration and invasion by regulating FTH.

### 2.6. The Loss of YTHDF1 Regulated FTH-Mediated Ferroptosis in Lung Cancer Cells

To assess whether the m^6^A reader YTHDF1 mediated ferroptosis by targeting FTH, we sought to determine whether the overexpression of FTH could reverse the alteration caused by YTHDF1 silencing. The results revealed that overexpressed FTH markedly suppressed intracellular total iron and Fe^2+^ levels (Figure 6A,B). Then, the productions of ROS and MDA were elevated upon the overexpression of FTH, while YTHDF1 silencing partially reversed the productions of ROS and MDA in both H1299 and A549 cells (Figure 6C,D). Last, a Western blot confirmed that YTHDF1 depletion reduced the level of GPX4, indicating that disruption of YTHDF1 promoted ferroptosis, while the overexpression of FTH could inverse the effect caused by YTHDF1 knockdown (Figure 6E,F). In conclusion, these results indicated that YTHDF1 might positively regulate FTH activity, as YTHDF1 deficiency induced FTH-mediated ferroptosis.

### 2.7. FTH Played an Oncogenic Role and Was Positively Correlated with YTHDF1 in Lung Cancer Patients

**Figure 7:**
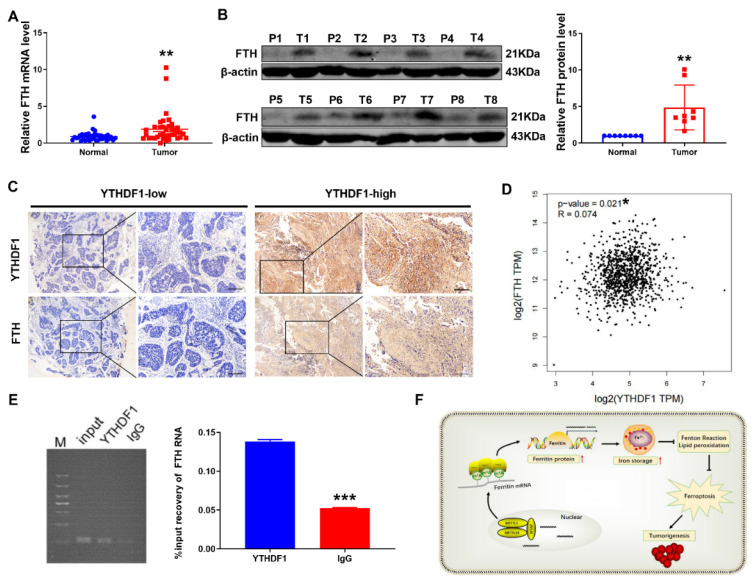
The expression of FTH is upregulated and positively correlated with YTHDF1 in lung cancer. (**A**,**B**) qRT-PCR and Western blot analysis of FTH mRNA expression and protein level in lung cancer tissues and adjacent normal tissues. (**C**) Representative immunohistochemical images of YTHDF1 and FTH in the lung cancer tissues. Scale bar, 50 μm. (**D**) FTH was shown to be markedly and negatively associated with YTHDF1 in the GEPIA database. (**E**) RIP was conducted to confirm the FTH mRNA enrichment by YTHDF1 in lung cancer tissues. (**F**) Schematic diagram indicating that YTHDF1 mediated ferritin in regulation of lung cancer carcinogenesis. ^**^ *p* < 0.01 and ^***^ *p* < 0.001.

In order to further investigate the role of FTH in the prognosis of lung cancer patients, the expression of FTH was detected by qRT-PCR and Western blots. The FTH mRNA expression and protein level were markedly elevated in lung cancer tissues compared with the normal tissues (Figure 7A,B). Furthermore, the relationship between YTHDF1 and FTH in lung cancer was investigated by immunohistochemistry. As expected, the expression of FTH showed similar upregulation with YTHDF1 in lung tumor tissues compared with para-tumor tissues (Figure 7C), which confirmed that inhibition of YTHDF1 could suppress FTH activity. Then, a Pearson correlation analysis was used to measure the relationship between YTHDF1 and FTH in lung cancer (Figure 7D). The results indicated that the expression of FTH was positively correlated with YTHDF1 expression in lung cancer patients (*p* = 0.021, Figure 7D), and also confirmed that YTHDF1 might regulate FTH expression in lung cancer patients. Furthermore, an RIP assay was executed to examine the association between YTHDF1 and FTH in lung cancer. The results suggested that the m^6^A-specific antibody significantly enriched FTH mRNA compared to the lgG control, revealing that the regulation of FTH level was under the control of YTHDF1-related m^6^A modification. To outline our findings, in lung cancer cells, elevated YTHDF1 activates ferritin translation and expression, thereby suppressing the production of intracellular iron metabolism and avoiding ferroptosis (Figure 7F).

## 3. Discussion

In our present study, we identified that YTHDF1 played a significant role in lung adenocarcinoma occurrence and metastasis, and YTHDF1 can be regarded as a predictable factor in lung cancer. The suppression of YTHDF1 could inhibit lung cancer cell proliferation and metastasis by decreasing the expression of FTH. FTH, as an iron metabolism relative protein, was identified as one of the possible target genes of YTHDF1. Further examination showed that FTH overexpression was sufficient to rescue the inhibitory effect of YTHDF1 knockdown on lung cancer cells. Furthermore, YTHDF1 depletion has been shown to decelerate the suppressive role of lung cancer cells by m^6^A methylation-mediated FTH downregulation. This evidence provided confirmation that the m^6^A–YTHDF1–FTH axis was crucially implicated in human lung carcinoma.

N6-methyladenosine (m^6^A), which contains “writers,” “erasers” and “readers”, was greatly related to types of tumor pathogenesis and progression [33]. Emerging evidence implies that abnormal amounts of m^6^A, as well as its related proteins, can affect key components [34], such as tumor suppressor and oncogene signaling pathways, ultimately impacting malignant cancer initiation and progression. YTHDF1 plays an essential role in different kinds of cancers and can increase the translation efficiency of m^6^A-modified mRNA in a cap-independent manner [35]. Several studies also reveal the complicated roles of the m^6^A modulator YTHDF1 in the pathological process of tumorigenesis and development. For instance, YTHDF1 affects translation of Frizzled7 in an m^6^A-dependent manner in gastric cancer [36]. Similarly, YTHDF1 promotes ARHGEF2 translation in an m^6^A-dependent manner and promotes progression of colorectal cancer through RhoA signaling [37]. Moreover, the oncogenic role of YTHDF1 was observed to influence the upregulation of RANBP2 mRNA and enhance the progression of cervical cancer in an m^6^A-dependent manner [38]. It is obvious that YTHDF1 regulates multiple signal pathways, but its exact mechanism on lung cancer has not been fully explored. In our research, we found that the expression of the m^6^A reader enzyme YTHDF1 in lung cancer was highly upregulated. In addition, the high expression of YTHDF1 in lung cancer patients had a relatively adverse prognosis. Then, we focused on the role of YTHDF1 in lung cancer cells and revealed the potential mechanism involved in this pathological process. As we expected, the preliminary experiments illustrated that the loss of YTHDF1 significantly inhibited lung cancer cell proliferation, migration and invasion, implying that it may act as an oncogene in the pathogenesis of lung cancer.

Ferroptosis, accompanied with iron-dependent reactive oxygen species (ROS) production and lipid peroxidation accumulation, was defined as a programmed cell death (PCD) [39]. Currently, there is a close connection between the risk of tumors and iron accumulation, while iron homeostasis dysfunction is considered a key metabolic “hallmark” of cancer [40,41,42]. Recently, several findings have shown that m^6^A is closely linked with ferroptosis in cancers [23,43]. However, it is unclear about the potential role between abnormal levels of m^6^A and ferroptosis in lung cancer. To further clarify the molecular mechanism of the m^6^A regulator YTHDF1, consistent with ferroptosis in lung carcinoma, we assessed intracellular Fe^2+^, intracellular ROS and lipid peroxidation levels in H1299 and A549 cells. We discovered that, in lung cancer cells, YTHDF1 depletion results in intracellular Fe^2+^ level increasing and iron accumulation, which causes ROS production and induces ferroptosis at last. Nevertheless, GPX4, as a critical regulator of ferroptosis [44], is notably downregulated after YTHDF1 silencing. Actually, the ferritin heavy subunit (FTH) is the major member of the iron storage protein that efficiently reduces the toxicity of Fe^2+^ and specifically recognizes ferroxidase activity, which is integral to the control of iron metabolism [45]. Notably, FTH plays a vital role in this process of cellular iron uptake and storage. Moreover, the disturbance of iron metabolism may also induce excessive intracellular iron storage and cause ferroptosis ultimately [46]. Hence, we further examined the potential mechanism between YTHDF1 and iron metabolism relative protein involved in ferroptosis. Indeed, we found that the lack of YTHDF1 inhibited FTH protein expression.

Methyltransferase-like 3 (METTL3) is a crucial protein in the methyltransferase complex, which mediates the degradation of target mRNA and plays a biological role in RNA modification [47]. Recently, studies have reported that METTL3 is highly expressed in bladder cancer and degrades SETD7 and KLF4 mRNAs combined with the m^6^A reader YTHDF2 [48]. Accordingly, in oral squamous cell carcinoma, METTL3 can collect the m^6^A reader YTHDF1 to facilitate their target c-MYC transcript stability [49]. In this finding, we further elucidated that m^6^A YTHDF1 mediates the translation of FTH increased and altered by disorder of METTL3 and, thereby, accelerates tumorigenesis in lung cancer cells, meaning FTH is an essential YTHDF1 target gene in lung cancer, obviously, in an m^6^A-dependent manner. Our subsequent assays confirmed that overexpression of FTH can sufficiently rescue the inhibitory effect of YTHDF1 silencing in cancer cell proliferation, migration and invasion, then leads to an adverse prognosis. Importantly, FTH overexpression also can reverse the constraint of YTHDF1 knockdown in lung cancer intracellular iron accumulation. Collectively, we found that the overexpression of FTH can restore gene expression and phenotypes induced by knockdown of YTHDF1, suggesting that FTH may be a main target to prevent lung carcinoma. Moreover, the expression of YTHDF1 and FTH were positively relevant in lung tissues, which indicated the clinical significance of YTHDF1-mediated m^6^A modification of FTH in lung adenocarcinoma occurrence and metastasis. An RIP assay also confirmed FTH mRNA enrichment by YTHDF1. In this study, we first identified the subtle relationship between FTH, ferroptosis and m^6^A modification in lung cancer cells and found that YTHDF1 could directly promote FTH mRNA translation in an m^6^A-dependent manner, but there are some limitations in our research. Based on in vitro study, we provided a preliminary discussion on the molecular mechanism of YTHDF1-mediated FTH translation in lung cancer cells, but our present study failed to investigate in vivo. Moreover, further studies are needed to identify the exact binding site about the regulatory role of YTHDF1 on FTH expression. In future research, we will contribute to addressing these unresolved problems, validating the exact molecular mechanisms of FTH in promoting the development and metastasis of lung cancer cells and regulating ferroptosis in vivo.

## 4. Materials and Methods

### 4.1. Tissue Samples

Samples of lung tumor and adjacent normal tissues were gathered from 29 patients with lung adenocarcinoma who had received curative surgery from the First Affiliated Hospital of Anhui Medical University. The study was approved by the Committee on Medical Ethics of the First Affiliated Hospital of Anhui Medical University (Permit number: Quick-PJ 2022-06-11, Date: 2022/05/26). Frozen tissues were subjected to detection of mRNA or protein expression using reverse transcription quantitative real-time PCR (qRT-PCR) or Western blotting analysis, and other tissues were applied for immunohistochemistry (IHC) analysis.

### 4.2. Immunohistochemistry Assay

Tumor tissues were fixed with 4% paraformaldehyde, then embedded in paraffin and cut into 5-µm-thick sections. Tumor tissues were deparaffinized and subjected to antigen recovery. After blocking with 10% normal goat serum for 60 min at 25 °C, the samples were incubated with anti-YTHDF1 (Proteintech, Wuhan, China) or anti-FTH (Absci, Washington, USA) at 4 °C overnight. The sections were continually incubated with HRP-labeled secondary antibody and stained using 3,3-diaminobenzidine (DAB). Hematoxylin was used for nuclear counterstaining. Finally, images were taken using a light microscope.

### 4.3. Cell Culture and Transfection

The non-small cell lung adenocarcinoma cell lines (H1299, A549) were purchased from the Fuheng Biotechnology Co., Ltd. (Shanghai, China) and cultured in 1640 medium supplemented with 10% fetal bovine serum (FBS). YTHDF1-specific siRNA transfections and negative control siRNAs were synthesized by Ribo and transfected into H1299 and A549 cells for 24 h using X-treme GENE Transfection Reagent. For the knockdown of YTHDF1, the cells were transfected with small interfering RNA of YTHDF1 or negative controls at a final concentration of 50 nM. For the overexpression of FTH, the cells were transfected with plasmids pcDNA3.1-FTH and negative controls at a final concentration of 500 ng/µL using Lipofectamine 3000 Transfection Reagent. The medium was changed every 6–8 h after transfection. Cells were used for protein/RNA extraction or immunofluorescent staining after transfection.

### 4.4. RNA Isolation and qRT-PCR

Trizol reagent was used to isolate the total RNA from clinical samples or cultured cell lines. Then, complementary deoxyribose nucleic acid (cDNA) was synthesized according to the instructions in the PrimeScriptTM RT MasterMix kit (Toyobo, Osaka, Japan). qRT-PCR was performed with SYBR Green qPCR Master Mix. The qRT-PCR primers were as follows: YTHDF1 forward 5′- GCACACAACCTCCATCTTCG-3′, YTHDF1 reverse 5′-AACTGGTTCGCCCTCATTGT-3′, FTH forward 5′- TGTGGCGGAGCTGCTGGGTAA-3′, FTH reverse 5′- CGAGAGGTGGATACGGCTGCT-3′, ACTIN forward 5′-CCTGGCACCCAGCACAAT-3′, ACTIN reverse 5′- GCTGATCCACATCTGCTGGAA -3′. Fold changes in mRNA expression were calculated using the 2−ΔΔCt method, and β-actin levels were taken for normalization.

### 4.5. Western Blot Analysis

The protein of clinical samples or cells was extracted by a radioimmunoprecipitation assay (RIPA) buffer supplemented with protease inhibitors. The bicinchoninic acid (BCA) protein assay kit was used to quantify protein concentration. The protein samples were mixed with SDS-PAGE loading buffer and heated in the heating block at 100 °C for 8 min. Approximately 50–100 μg of protein samples was separated using 10–12% SDS-PAGE and transferred to NC membranes. After blocking using 5% nonfat milk for 2 h, the membranes were immune-stained with primary antibodies YTHDF1 (1:2000; Proteintech, Wuhan, China), FTH (1:500; Absci, Washington, USA), GPX4 (1:2000; abcam, Cambridge, UK) and β-actin (1:2000; Proteintech, Wuhan, China) at 4 °C overnight. After washing three times in PBS containing Tween 20 (PBST), the membranes were incubated with a secondary antibody for 50 min at room temperature. Band signals were detected using the imaging system and analyzed using Image Studio 5.2 Software. β-actin was used as the internal reference to quantitate relative protein expression levels.

### 4.6. Cell Viability Assay

The cell viability was measured by a cell counting kit-8 assay. Pretreated cells were counted and seeded into a 96-well plate at a density of 2 × 103 cells/well. CCK-8 reagent was added and incubated at 37 °C for 1.5 h after 24, 48, 72 and 96 h, then the optical density (OD) value was detected at 450 nm.

### 4.7. Edu Incorporation Assay

Cell proliferation capacity was measured by a 5-ethynyl-2-deoxyuridine (EdU) incorporation assay kit. Pretreated cells were treated with 50 μM EdU for 2 h at 37 °C. Next, the cells were fixed with 4% paraformaldehyde for 15 min and permeabilized with 0.5% Triton X-100 for 10 min. In each well was added 100 μL click reaction solution for 30 min and the nuclei were stained with Hoechst 33342. The images were viewed and captured under a fluorescence microscope.

### 4.8. Colony-Formation Assay

The transfected cells were counted and seeded into 6-well plates (800 cells/well). The cells were in standardized culture for 1 or 2 weeks and the culture medium was replaced every 2–3 days. After 30 min of fixation with paraformaldehyde, colonies were washed with PBS and stained with 0.1% crystal violet for 30 min. Then, the colony counts and rate were calculated and analyzed by GraphPad prism 7.0.

### 4.9. Wound-Healing Assay

Pretreated cells were cultured overnight in complete medium to reach the confluence. The cell layers were scratched by using sterile 200 µL pipette tips and replaced with fresh medium. Wound-healing status was imaged at 0 and 48 h after scratching. The wound area was analyzed using Image J software v1.8.0.

### 4.10. Transwell Assay

The cell invasion capacity was revealed by the Transwell assay. Transfected cells (1 × 105 H1299 cells and 1 × 105 A549 cells) were seeded into the upper chambers with serum-free medium, which was coated with Matrigel for the invasion assays. Medium containing 10% FBS was added to the bottom chambers. After incubation for 48 h, the medium was removed and then cells were fixed with 4% paraformaldehyde for 30 min and stained by 0.1% crystal violet for 30 min. The invasive cells were counted on a light microscope.

### 4.11. Iron Assay

The intracellular ferrous iron level (Fe^2+^) was detected by using the iron assay kit. Firstly, samples were collected, washed with PBS and homogenized in iron assay buffer, then the standard, test samples and iron standards containing iron reducer were added and incubated at 37 °C for 30 min. Finally, an iron probe was added. The mixture was incubated for 60 min and then read at 593 nm by SoftMax® Pro 7 Software for detections.

### 4.12. ROS Detection

The total cellular ROS level was detected by using 2′,7′-Dichlorofluorescin diacetate (DCFDA) dye from an ROS assay kit according to the manufacturer’s instructions. A total of 10 μM DCFH-DA was added into the pretreated cells for detection, and cells were incubated at 37 °C for 20 min. The ROS level in the cells was observed by a fluorescence microscope.

### 4.13. Lipid Peroxidation Assay

Lipid peroxidation was evaluated by the Lipid Peroxidation Assay Kit (BiYunTian Biotechnology, Shanghai, China). The cell lysate supernatants were collected, and MDA concentration was added. The cells were incubated at 100 °C for 15 min, and the level of MDA was measured by a SpectraMax^®^ i3x Plate Reader at 532 nm.

### 4.14. RNA Immune Precipitation (RIP)

The indicated tissue lysates were collected and the supernatant complexes containing RNA proteins were then treated for 30 min at room temperature with 5 μg of control IgG antibody and YTHDF1 antibody (Proteintech, Wuhan, China) separately. Each tube was filled with 900 μL of RIP Immunoprecipitation Buffer, then 100 μL of the supernatant was pipetted into the magnetic bead–antibody complex and incubated at 4 °C for 3 h to overnight. The beads were washed four times with RIP Wash Buffer after being gathered on a magnetic platform. The next day, the magnetic bead–antibody combination was resuspended in 150 μL of Proteinase K Buffer and incubated for 30 min at 55 °C. Following RNA purification, reverse transcription was carried out for qPCR detection and analysis.

### 4.15. Gene Expression and Coefficient Analysis in Lung Cancer Datasets

Several user-friendly databases or tools were utilized to analyze YTHDF1 and FTH expression levels in lung cancer, and the Cancer Genome Atlas (TCGA) database [50] was employed to measure the difference of YTHDF1 expression between lung cancer and matched normal samples. The associated integrated database was used to assess and analyze correlation between the expression of YTHDF1 and FTH in lung cancer samples.

### 4.16. Statistical Analysis

Statistical analyses were performed using GraphPad Prism 7.0. All results were expressed as mean ± standard error of the mean (SEM). A t-test was adopted for comparison between groups and a one-way ANOVA was used for comparisons among multiple groups. A Pearson’s correlation coefficient analysis was used for analyzing correlations. *p* < 0.05 indicated statistically significant differences.

## 5. Conclusions

In summary, we found that the aberrated regulation of the m^6^A modification reader YTHDF1 in lung cancer caused tumorigenesis and metastasis, further revealed that YTHDF1 promotes the growth and metastasis of lung cancer cells via upregulating FTH translation and protein expression and identified FTH as a downstream target of YTHDF1-mediated m^6^A modification. Given the widespread finding that YTHDF1 is upregulated and acts as a prognostic biomarker in lung adenocarcinoma progression, targeting the YTHDF1–m^6^A–FTH axis could be a promising therapeutic strategy for inhibition of lung cancer occurrence and development. Overall, our research not only provides a fresh novel insight into the lung cancer pathogenesis about the specific molecular mechanism, but also paves the way for the development of more effective therapeutic strategies for lung cancer.

## Data Availability

The datasets used and/or analyzed in the current study are available from the corresponding author upon reasonable request.

## References

[1] Bray F., Ferlay J., Soerjomataram I., Siegel R.L., Torre L.A., Jemal A. (2018). Global cancer statistics 2018: GLOBOCAN estimates of incidence and mortality worldwide for 36 cancers in 185 countries. CA Cancer J. Clin..

[2] Barnet M.B., Cooper W.A., Boyer M.J., Kao S. (2018). Immunotherapy in Non-Small Cell Lung Cancer: Shifting Prognostic Paradigms. J. Clin. Med..

[3] Suzuki S., Goto T. (2020). Role of Surgical Intervention in Unresectable Non-Small Cell Lung Cancer. J. Clin. Med..

[4] Arbour K.C., Riely G.J. (2019). Systemic Therapy for Locally Advanced and Metastatic Non-Small Cell Lung Cancer: A Review. JAMA.

[5] Cao G., Li H.B., Yin Z., Flavell R.A. (2016). Recent advances in dynamic m6A RNA modification. Open Biol..

[6] Wang X., Lu Z., Gomez A., Hon G.C., Yue Y., Han D., Fu Y., Parisien M., Dai Q., Jia G. (2014). N6-methyladenosine-dependent regulation of messenger RNA stability. Nature.

[7] Wang X., Zhao B.S., Roundtree I.A., Lu Z., Han D., Ma H., Weng X., Chen K., Shi H., He C. (2015). N(6)-methyladenosine Modulates Messenger RNA Translation Efficiency. Cell.

[8] Deng X., Su R., Weng H., Huang H., Li Z., Chen J. (2018). RNA N(6)-methyladenosine modification in cancers: Current status and perspectives. Cell Res..

[9] Chen Y., Peng C., Chen J., Chen D., Yang B., He B., Hu W., Zhang Y., Liu H., Dai L. (2019). WTAP facilitates progression of hepatocellular carcinoma via m6A-HuR-dependent epigenetic silencing of ETS1. Mol. Cancer.

[10] Liu X., Qin J., Gao T., Li C., He B., Pan B., Xu X., Chen X., Zeng K., Xu M. (2020). YTHDF1 Facilitates the Progression of Hepatocellular Carcinoma by Promoting FZD5 mRNA Translation in an m6A-Dependent Manner. Mol. Ther. Nucleic Acids.

[11] Li T., Hu P.S., Zuo Z., Lin J.F., Li X., Wu Q.N., Chen Z.H., Zeng Z.L., Wang F., Zheng J. (2019). METTL3 facilitates tumor progression via an m(6)A-IGF2BP2-dependent mechanism in colorectal carcinoma. Mol. Cancer.

[12] Jin H., Ying X., Que B., Wang X., Chao Y., Zhang H., Yuan Z., Qi D., Lin S., Min W. (2019). N(6)-methyladenosine modification of ITGA6 mRNA promotes the development and progression of bladder cancer. EBioMedicine.

[13] Niu Y., Lin Z., Wan A., Chen H., Liang H., Sun L., Wang Y., Li X., Xiong X.F., Wei B. (2019). RNA N6-methyladenosine demethylase FTO promotes breast tumor progression through inhibiting BNIP3. Mol. Cancer.

[14] Chen Z., Zhong X., Xia M., Zhong J. (2021). The roles and mechanisms of the m6A reader protein YTHDF1 in tumor biology and human diseases. Mol. Ther. Nucleic Acids.

[15] Li Q., Ni Y., Zhang L., Jiang R., Xu J., Yang H., Hu Y., Qiu J., Pu L., Tang J. (2021). HIF-1alpha-induced expression of m6A reader YTHDF1 drives hypoxia-induced autophagy and malignancy of hepatocellular carcinoma by promoting ATG2A and ATG14 translation. Signal Transduct. Target Ther..

[16] Cao J.Y., Dixon S.J. (2016). Mechanisms of ferroptosis. Cell Mol. Life Sci..

[17] Dixon S.J., Stockwell B.R. (2014). The role of iron and reactive oxygen species in cell death. Nat. Chem. Biol..

[18] Tang B., Zhu J., Li J., Fan K., Gao Y., Cheng S., Kong C., Zheng L., Wu F., Weng Q. (2020). The ferroptosis and iron-metabolism signature robustly predicts clinical diagnosis, prognosis and immune microenvironment for hepatocellular carcinoma. Cell Commun. Signal.

[19] Park E., Chung S.W. (2019). ROS-mediated autophagy increases intracellular iron levels and ferroptosis by ferritin and transferrin receptor regulation. Cell Death Dis..

[20] Hou W., Xie Y., Song X., Sun X., Lotze M.T., Zeh H.J., Kang R., Tang D. (2016). Autophagy promotes ferroptosis by degradation of ferritin. Autophagy.

[21] Orino K., Lehman L., Tsuji Y., Ayaki H., Torti S.V., Torti F.M. (2001). Ferritin and the response to oxidative stress. Biochem. J..

[22] Ye J., Wang Z., Chen X., Jiang X., Dong Z., Hu S., Li W., Liu Y., Liao B., Han W. (2020). YTHDF1-enhanced iron metabolism depends on TFRC m(6)A methylation. Theranostics.

[23] Fan Z., Yang G., Zhang W., Liu Q., Liu G., Liu P., Xu L., Wang J., Yan Z., Han H. (2021). Hypoxia blocks ferroptosis of hepatocellular carcinoma via suppression of METTL14 triggered YTHDF2-dependent silencing of SLC7A11. J. Cell. Mol. Med..

[24] Shen M., Li Y., Wang Y., Shao J., Zhang F., Yin G., Chen A., Zhang Z., Zheng S. (2021). N(6)-methyladenosine modification regulates ferroptosis through autophagy signaling pathway in hepatic stellate cells. Redox Biol..

[25] Stockwell B.R., Friedmann Angeli J.P., Bayir H., Bush A.I., Conrad M., Dixon S.J., Fulda S., Gascon S., Hatzios S.K., Kagan V.E. (2017). Ferroptosis: A Regulated Cell Death Nexus Linking Metabolism, Redox Biology, and Disease. Cell.

[26] Roundtree I.A., Evans M.E., Pan T., He C. (2017). Dynamic RNA Modifications in Gene Expression Regulation. Cell.

[27] Salatino A., Aversa I., Battaglia A.M., Sacco A., Di Vito A., Santamaria G., Chirillo R., Veltri P., Tradigo G., Di Cello A. (2019). H-Ferritin Affects Cisplatin-Induced Cytotoxicity in Ovarian Cancer Cells through the Modulation of ROS. Oxid. Med. Cell Longev..

[28] Tian Y., Lu J., Hao X., Li H., Zhang G., Liu X., Li X., Zhao C., Kuang W., Chen D. (2020). FTH1 Inhibits Ferroptosis Through Ferritinophagy in the 6-OHDA Model of Parkinson’s Disease. Neurotherapeutics.

[29] Wang Q., Guo X., Li L., Gao Z., Su X., Ji M., Liu J. (2020). N(6)-methyladenosine METTL3 promotes cervical cancer tumorigenesis and Warburg effect through YTHDF1/HK2 modification. Cell Death Dis..

[30] He Y., Wang W., Xu X., Yang B., Yu X., Wu Y., Wang J. (2022). Mettl3 inhibits the apoptosis and autophagy of chondrocytes in inflammation through mediating Bcl2 stability via Ythdf1-mediated m(6)A modification. Bone.

[31] Xu Y., Lv D., Yan C., Su H., Zhang X., Shi Y., Ying K. (2022). METTL3 promotes lung adenocarcinoma tumor growth and inhibits ferroptosis by stabilizing SLC7A11 m(6)A modification. Cancer Cell Int..

[32] Zhao Y., Shi Y., Shen H., Xie W. (2020). m(6)A-binding proteins: The emerging crucial performers in epigenetics. J. Hematol. Oncol..

[33] Yang Y., Hsu P.J., Chen Y.S., Yang Y.G. (2018). Dynamic transcriptomic m(6)A decoration: Writers, erasers, readers and functions in RNA metabolism. Cell Res..

[34] Lin X., Chai G., Wu Y., Li J., Chen F., Liu J., Luo G., Tauler J., Du J., Lin S. (2019). RNA m(6)A methylation regulates the epithelial mesenchymal transition of cancer cells and translation of Snail. Nat. Commun..

[35] Zhang Y., Liu X., Liu L., Li J., Hu Q., Sun R. (2020). Expression and Prognostic Significance of m6A-Related Genes in Lung Adenocarcinoma. Med. Sci. Monit..

[36] Pi J., Wang W., Ji M., Wang X., Wei X., Jin J., Liu T., Qiang J., Qi Z., Li F. (2021). YTHDF1 Promotes Gastric Carcinogenesis by Controlling Translation of FZD7. Cancer Res..

[37] Wang S., Gao S., Zeng Y., Zhu L., Mo Y., Wong C.C., Bao Y., Su P., Zhai J., Wang L. (2022). N6-Methyladenosine Reader YTHDF1 Promotes ARHGEF2 Translation and RhoA Signaling in Colorectal Cancer. Gastroenterology.

[38] Wang H., Luo Q., Kang J., Wei Q., Yang Y., Yang D., Liu X., Liu T., Yi P. (2021). YTHDF1 Aggravates the Progression of Cervical Cancer Through m(6)A-Mediated Up-Regulation of RANBP2. Front. Oncol..

[39] Imai H., Matsuoka M., Kumagai T., Sakamoto T., Koumura T. (2017). Lipid Peroxidation-Dependent Cell Death Regulated by GPx4 and Ferroptosis. Curr. Top. Microbiol. Immunol..

[40] Zhou L., Zhao B., Zhang L., Wang S., Dong D., Lv H., Shang P. (2018). Alterations in Cellular Iron Metabolism Provide More Therapeutic Opportunities for Cancer. Int. J. Mol. Sci..

[41] Chen Z., Wu L., Zhou J., Lin X., Peng Y., Ge L., Chiang C.M., Huang H., Wang H., He W. (2020). N6-methyladenosine-induced ERRgamma triggers chemoresistance of cancer cells through upregulation of ABCB1 and metabolic reprogramming. Theranostics.

[42] Zhang Y., Kong Y., Ma Y., Ni S., Wikerholmen T., Xi K., Zhao F., Zhao Z., Wang J., Huang B. (2021). Loss of COPZ1 induces NCOA4 mediated autophagy and ferroptosis in glioblastoma cell lines. Oncogene.

[43] Zou Y., Zheng S., Xie X., Ye F., Hu X., Tian Z., Yan S., Yang L., Kong Y., Tang Y. (2022). N6-methyladenosine regulated FGFR4 attenuates ferroptotic cell death in recalcitrant HER2-positive breast cancer. Nat. Commun..

[44] Yang W.S., SriRamaratnam R., Welsch M.E., Shimada K., Skouta R., Viswanathan V.S., Cheah J.H., Clemons P.A., Shamji A.F., Clish C.B. (2014). Regulation of ferroptotic cancer cell death by GPX4. Cell.

[45] Munro H.N. (1990). Iron regulation of ferritin gene expression. J. Cell Biochem..

[46] Blankenhaus B., Braza F., Martins R., Bastos-Amador P., Gonzalez-Garcia I., Carlos A.R., Mahu I., Faisca P., Nunes J.M., Ventura P. (2019). Ferritin regulates organismal energy balance and thermogenesis. Mol. Metab..

[47] Lin S., Choe J., Du P., Triboulet R., Gregory R.I. (2016). The m(6)A Methyltransferase METTL3 Promotes Translation in Human Cancer Cells. Mol. Cell.

[48] Xie H., Li J., Ying Y., Yan H., Jin K., Ma X., He L., Xu X., Liu B., Wang X. (2020). METTL3/YTHDF2 m(6) A axis promotes tumorigenesis by degrading SETD7 and KLF4 mRNAs in bladder cancer. J. Cell Mol. Med..

[49] Zhao W., Cui Y., Liu L., Ma X., Qi X., Wang Y., Liu Z., Ma S., Liu J., Wu J. (2020). METTL3 Facilitates Oral Squamous Cell Carcinoma Tumorigenesis by Enhancing c-Myc Stability via YTHDF1-Mediated m(6)A Modification. Mol. Ther. Nucleic Acids.

[50] Tang Z., Li C., Kang B., Gao G., Li C., Zhang Z. (2017). GEPIA: A web server for cancer and normal gene expression profiling and interactive analyses. Nucleic Acids Res..

